# An infinite two-dimensional hybrid water–chloride network in a 4′-(furan-2-yl)-2,2′:6′,2′′-terpyridine nickel(II) matrix

**DOI:** 10.1107/S2056989017007095

**Published:** 2017-05-23

**Authors:** Wei-Wei Fu, Ya-Qian Li, Yang Liu, Man-Sheng Chen, Wei Li, Ying-Qun Yang

**Affiliations:** a Key Laboratory of Functional Organometallic Materials of General Colleges and Universities in Hunan Province, Department of Chemistry and Materials Science, Hengyang Normal University, Hengyang 421008, People’s Republic of China

**Keywords:** crystal structure, terpyridine, Ni^II^, water cluster, water–chloride network, hydrogen bonds

## Abstract

A new complex, namely bis­[4′-(furan-2-yl)-2,2′:6′,2′′-terpyridine]­nickel(II) dichloride deca­hydrate, [Ni(C_19_H_13_N_3_O)_2_]Cl_2_·10H_2_O, has been crystallized by solvent evaporation and characterized by single-crystal X-ray diffraction. The coordination environment of the Ni^II^ cation is distorted octa­hedral with slight deviations from an idealized geometry. The most intriguing structural feature is an infinite two-dimensional hybrid water–chloride network parallel to (011) constructed by O—H⋯O and O—H⋯Cl hydrogen bonds involving two independent chloride ions and ten independent solvent water mol­ecules with an l-shaped pattern. One of the furyl rings is disordered with a refined occupancy ratio of 0.786 (13):0.214 (13)

## Abstract 2

An unprecedented two-dimensional water–chloride anionic {[(H_2_O)_10_Cl_2_]^2−^}_*n*_ network has been structurally identified in a hydro­phobic matrix of the nickel(II) complex [Ni(ftpy)_2_]Cl_2_·10H_2_O [ftpy = 4′-(furan-2-yl)-2,2′:6′,2′′-terpyridine].

## Chemical context   

Water has received much scientific inter­est as it is a major chemical constituent on the earth’s surface and it is also the source of life. Many discrete water clusters and polymeric water aggregates, with different types of hydrogen bonds and in diverse sizes and shapes, captured in the crystal lattice of an organic or metal coordination complex during crystallization have been found and investigated experimentally and theoretically (Dutta *et al.*, 2015 ▸; Ganguly & Mondal, 2015 ▸; Han *et al.*, 2014 ▸; Hundal *et al.*, 2014 ▸; Pati *et al.*, 2014 ▸).
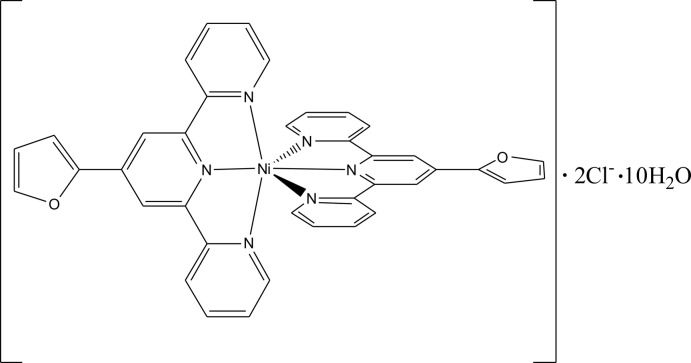


**Figure 1:**
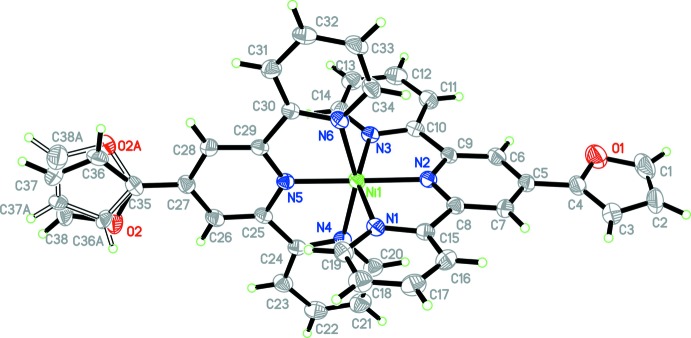
The mol­ecular structure of [Ni(ftpy)_2_]^2+^ in **1**, with displacement ellipsoids drawn at the 30% probability level.

Hybrid water–chloride associates incorporated in various crystal matrixes are one of the most inter­esting combinations in water clusters research due to their fundamental importance for understanding water–halide inter­actions in the atmosphere, the ocean and in biological systems (Inumaru *et al.*, 2008 ▸; Kumar *et al.*, 2011 ▸; Lakshminarayanan *et al.*, 2006 ▸; Li *et al.*, 2008 ▸). According to a search of the Cambridge Structural Database (CSD Version 5.37, May 2016; Groom *et al.*, 2016 ▸), there are about nine examples with water–chloride hydrogen bonds forming one-dimensional tapes (Boyer *et al.*, 2011 ▸; van Holst *et al.*, 2008 ▸; Kepert *et al.*, 1999 ▸; Jitsukawa *et al.*, 1994 ▸), two-dimensional (Kepert *et al.*, 1994 ▸; Chowdhury *et al.*, 2011 ▸; Duan *et al.*, 2016 ▸) and three-dimensional (Figgis *et al.*, 1983 ▸; Pruchnik *et al.*, 1996 ▸) networks from 2,2′:6′,2′′-terpyridine ligands. When 4′-substituted terpyridines with phenyl, pyridyl, imidazolyl rings were considered, two-dimensional and three-dimensional water–chloride networks with two chloride ions and at least six water mol­ecules were found (Constable *et al.*, 1990 ▸; Kou *et al.*, 2008 ▸; Chen *et al.*, 2013 ▸; Fernandes *et al.*, 2008 ▸; McMurtrie & Dance, 2010 ▸; Padhi *et al.*, 2010 ▸; Indumathy *et al.*, 2008 ▸; Mahendiran *et al.*, 2016 ▸). The hydro­phobic and hydro­philic layers are further linked by two kinds of C—H⋯O hydrogen bonds into three-dimensional networks. In this context, a ftpy–Ni^II^ complex [ftpy = 4′-(furan-2-yl)-2,2′:6′,2′′-terpyridine] (Fig. 1 ▸) with two chlorides as counter-ions and ten solvent water mol­ecules (**1**) is described herein.

## Structural commentary   

The asymmetric unit of **1** is composed of a cationic [Ni(ftpy)_2_]^2+^ part, two chloride anions, and ten water mol­ecules of crystallization. The distances between Ni1 and the N atoms of the central pyridyl rings [1.974 (3) and 1.977 (3) Å] are slightly shorter than those between Ni1 and the N atoms of outer pyridyl rings [2.093 (3) −2.099 (3) Å; Table 1 ▸]. The angles involving Ni1 can be divided into two sets, *viz*. three transoid angles [178.36 (10), 155.38 (11) and 155.89 (11)°] and 12 cisoid angles, which range from 77.74 (11) to 103.80 (10)°. The differences in the bond lengths and angles indicate a distorted octa­hedral geometry (Constable *et al.*, 1990 ▸; Logacheva *et al.*, 2009 ▸; Padhi *et al.*, 2010 ▸; Fu *et al.*, 2013 ▸). The terpyridyl ring systems [maximum deviations of ±0.058 (4) Å for C27/C31 and 0.192 (4) Å for C17] are almost perpendicular to each other, subtending a dihedral angle of 87.35 (6)°. The furyl rings are almost coplanar with the terpyridyl ring systems, making dihedral angles of 8.1 (2) and 3.2 (3)° for the O1- and O2-containing rings, respectively.

## Supra­molecular features   

**Figure 2:**
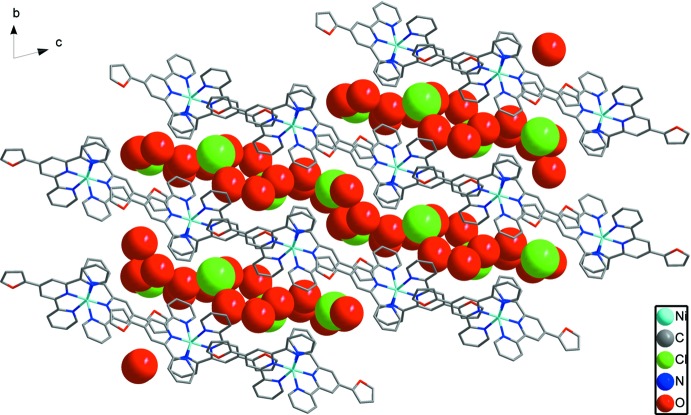
View of the hydro­phobic (represented by wireframes) and hydro­philic (represented by spheres) layers in **1**.

**Figure 3:**
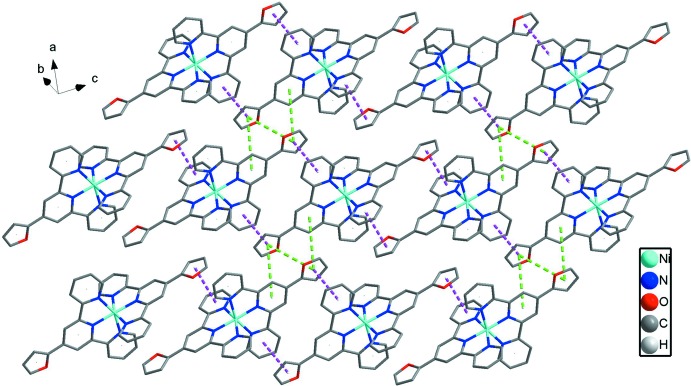
A view of the two-dimensional undulating sheet of hydro­phobic layers, with π–π inter­actions highlighted by dashed lines [purple for 3.533 (5) and 3.761 (4) Å, and green for 4.338 (14) and 4.405 (4) Å].

**Figure 4:**
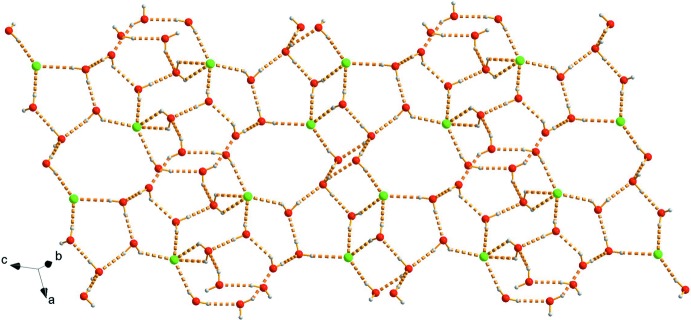
A view of the hybrid water–chloride hydrogen-bonded assemblies in **1**, with water mol­ecules and chloride anions shown as coloured balls and hydrogen bonds as dashed lines.

In the crystal, there are hydro­phobic layers composed of [Ni(ftpy)_2_]^2+^ dications and hydro­philic layers composed of water mol­ecules and chloride anions (Fig. 2 ▸). In the hydro­phobic layers, shown in Fig. 3 ▸, [Ni(ftpy)_2_]^2+^ dications are linked by two kinds of face-to-face π–π inter­actions with centroid–centroid distances of 3.530 (4) and 3.760 (4) Å between the furyl and outer pyridyl rings, forming one-dimensional (1D) chains. These 1D chains are linked by further π–π inter­actions with centroid distances of 4.367 (4) Å between furyl rings and 4.405 (4) Å between furyl and central pyridyl rings, forming two-dimensional networks. The water mol­ecules and chloride anions form a two-dimensional network parallel to (011) *via* O—H⋯O and O—H⋯Cl hydrogen bonds (Table 2 ▸), as shown in Fig. 4 ▸.

**Figure 5:**
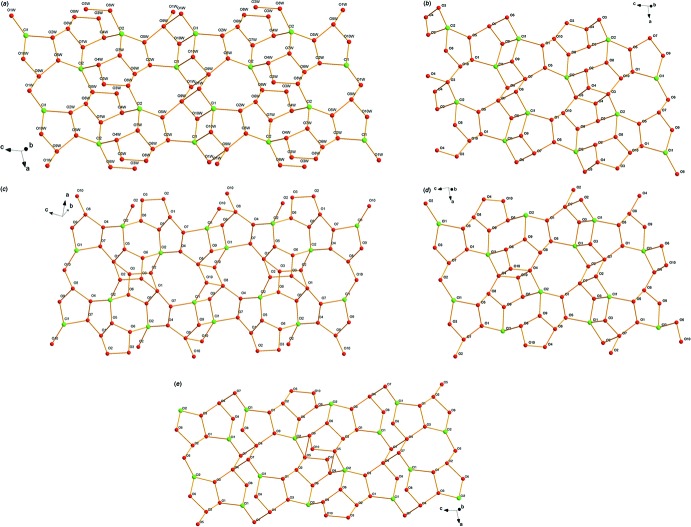
Multicyclic {[(H_2_O)_10_Cl_2_]^2−^}_*n*_ fragments with repeating units of two-dimensional water–chloride networks in (*a*) **1**, (*b*) **2**, (*c*) **3**, (*d*) **4** and (*e*) **5**.

The multicyclic {[(H_2_O)_10_Cl_2_]^2−^}_*n*_ fragments in the hydro­philic layers are constructed by means of 11 non-equivalent O—H⋯O hydrogen bonds with O⋯O distances ranging from 2.756 (6) to 3.134 (7) Å and nine O—H⋯Cl hydrogen bonds with O⋯Cl distances ranging from 3.079 (4) to 3.225 (4) Å (Table 2 ▸, Fig. 4 ▸). Both the O⋯O and O⋯Cl distances are comparable with those found in various types of water clusters and water–chloride associates (Safin *et al.*, 2015 ▸; Bhat & Revankar, 2016 ▸; Ris *et al.*, 2016 ▸). The resulting two-dimensional network can be considered as a set of alternating cyclic fragments with three tetra­nuclear, three penta­nuclear, one hexa­nuclear and two octa­nuclear fragments, as shown in Fig. 5 ▸ *a*. Two of these fragments are composed only of water mol­ecules, whereas the other seven rings are water–chloride hybrids with one or two Cl^−^ anions. Most of the rings are non-planar, contributing to the formation of an intricate relief geometry of the water–chloride layer. Using the method described by Infantes and co-workers (Infantes & Motherwell, 2002 ▸; Infantes *et al.*, 2003 ▸), this two-dimensional water–chloride network can be described as having an *L*4(6)4(6)4(6)5(5)5(6)5(6)6(8)8(8)8(10) pattern.

## Comparison with other terpyridine complexes possessing 10 solvent water mol­ecules   

It is inter­esting to make a comparison of the two-dimensional water–chloride networks in **1** and those found in other terpyridine complexes possessing 10 solvent water mol­ecules, *viz*. [Fe(phtpy)_2_]Cl_2_·10H_2_O (**2**; refcode: VOBKON; Fernandes *et al.*, 2008 ▸), [Ni(phtpy)_2_]Cl_2_·10H_2_O, (**3**; refcode: SIXLIU01; Chen *et al.*, 2013 ▸), [Ru(phtpy)_2_]Cl_2_·10H_2_O (**4**; refcode: FAFFID; McMurtrie & Dance, 2010 ▸) and [Ru(pytpy)_2_]Cl_2_·10H_2_O (**5**; refcode: TUXGUP; Padhi *et al.*, 2010 ▸) [phtpy = 4′-phenyl-2,2′:6′,2′′-terpyridine and pytpy = 4′-(2-pyrid­yl)-2,2′:6′,2′′-terpyridine]. In spite of the differences in the metal ions and terpyridine ligands, the crystal parameters are almost the same for compounds **2**–**5**. Where a five-membered furyl ring is involved instead of a six-membered phenyl or pyridyl ring, the size of the crystal cell decreases with reduction in the cell volume of about 4.5% from 2200 to 2100 Å^3^. Considering the O⋯O and O⋯Cl distances within the two-dimensional water–chloride networks, a different number of trinuclear, tetra­nuclear, penta­nuclear, hexa­nuclear and octa­nuclear rings have been determined, giving an *L*4(6)4(6)4(6)4(6)4(6)5(6)5(6)5(6)6(8)8(12) pattern for **2**, an *L*4(6)4(6)4(6)5(7)5(7)5(8)5(8)6(7)6(9)6(9)8(12) pattern for **3**, an *L*4(6)4(6)4(6)4(6)4(6)4(6)5(6)5(6)5(7)6(7)8(12) pattern for **4** and an *L*3(6)4(6)5(5)5(6)5(6)6(8)6(8)8(8)8(10) pattern for **5** (Fig. 5 ▸ *b*–*e*). These results illustrate how a water–chloride assembly could be fine-tuned by adopting diverse ligands and different metal ions. It is potentially useful for future studies of water–water or water–chloride inter­actions for chemists as well as theoreticians.

## Synthesis and crystallization   

4′-Furyl-2,2′:6′,2′′-terpyridine was prepared by a literature method (Wang & Hanan, 2005 ▸). Other reagents and solvents used in reactions were purchased from Aladdin Chemical and used without purification, unless otherwise indicated.

NiCl_2_·6H_2_O (0.1 mmol, 0.024g) and ftpy (0.2 mmol, 0.060 g) were dissolved in 10 ml distilled water and 10 ml methanol. The solution was left alone for slow evaporation without disturbance for about one month and reddish brown crystals of (**1**) suitable for X-ray analysis were obtained.

## Refinement   

Crystal data, data collection and structure refinement details are summarized in Table 3 ▸. All hydrogen atoms except those of water mol­ecules were generated geometrically and refined isotropically using a riding model, with C—H = 0.93 Å and *U*_iso_(H) = 1.2*U*_eq_(C). The hydrogen atoms of solvent water mol­ecules were located in difference-Fourier maps, refined with DFIX restraints of O—H distances and finally fixed at those positions using AFIX 3 in *SHELXL* (Sheldrick, 2015*b* ▸). Atoms C36, C37, C38 and O2 were found to be disordered over two sets of sites with a refined occupancy ratio of 0.786 (13):0.214 (13) for C36/C36*A*, C37/C37*A*, C38/C38*A*, and O2/O2*A*. In order to model the disorder of this furyl ring, various restraints (DFIX, FLAT, ISOR, DELU, EADP) were applied in the refinement.

## Supplementary Material

Crystal structure: contains datablock(s) I. DOI: 10.1107/S2056989017007095/lh5845sup1.cif

Structure factors: contains datablock(s) I. DOI: 10.1107/S2056989017007095/lh5845Isup2.hkl

CCDC reference: 1498201

Additional supporting information:  crystallographic information; 3D view; checkCIF report

## Figures and Tables

**Table 1:** Selected geometric parameters (Å, °)

Ni1—N5	1.974 (3)	Ni1—N3	2.096 (3)
Ni1—N2	1.977 (3)	Ni1—N1	2.098 (3)
Ni1—N6	2.093 (3)	Ni1—N4	2.099 (3)
			
N5—Ni1—N2	178.36 (10)	N2—Ni1—N1	77.77 (11)
N5—Ni1—N6	77.81 (11)	N6—Ni1—N1	93.13 (11)
N2—Ni1—N6	102.65 (11)	N3—Ni1—N1	155.38 (11)
N5—Ni1—N3	100.71 (11)	N5—Ni1—N4	78.10 (11)
N2—Ni1—N3	77.74 (11)	N2—Ni1—N4	101.46 (12)
N6—Ni1—N3	89.84 (11)	N6—Ni1—N4	155.89 (11)
N5—Ni1—N1	103.80 (10)	N3—Ni1—N4	95.46 (11)

**Table 2:** Hydrogen-bond geometry (Å, °)

*D*—H⋯*A*	*D*—H	H⋯*A*	*D*⋯*A*	*D*—H⋯*A*
O1*W*—H1*WA*⋯Cl1	0.87	2.25	3.113 (4)	169
O1*W*—H1*WB*⋯O9*W* ^i^	0.87	2.06	2.923 (6)	175
O2*W*—H2*WB*⋯O5*W* ^ii^	0.83	1.99	2.813 (7)	172
O2*W*—H2*WA*⋯Cl1	0.84	2.39	3.215 (4)	168
O3*W*—H3*WC*⋯O4*W*	0.86	2.05	2.760 (9)	140
O3*W*—H3*WA*⋯O6*W* ^iii^	0.88	2.35	3.134 (7)	148
O4*W*—H4*WB*⋯Cl2	0.88	2.58	3.107 (5)	119
O4*W*—H4*WA*⋯Cl2	0.87	2.56	3.107 (5)	122
O5*W*—H5*WA*⋯Cl2	0.87	2.37	3.079 (4)	138
O5*W*—H5*WB*⋯O9*W*	0.89	2.16	2.991 (6)	156
O6*W*—H6*WC*⋯O2*W* ^ii^	0.83	2.11	2.929 (6)	167
O6*W*—H6*WA*⋯O7*W*	0.83	2.18	2.838 (6)	136
O7*W*—H7*WA*⋯Cl2	0.87	2.34	3.190 (4)	167
O7*W*—H7*WB*⋯O4*W* ^ii^	0.87	1.93	2.798 (5)	172
O8*W*—H8*WC*⋯O3*W* ^ii^	0.85	2.06	2.856 (8)	155
O8*W*—H8*WD*⋯Cl2^iv^	0.85	2.40	3.204 (6)	157
O9*W*—H9*WA*⋯O10*W* ^v^	0.86	1.93	2.756 (6)	159
O9*W*—H9*WB*⋯O1*W* ^vi^	0.86	2.11	2.878 (5)	147
O10*W*—H10*A*⋯Cl1^vii^	0.88	2.27	3.141 (4)	171
O10*W*—H10*B*⋯Cl1^viii^	0.87	2.38	3.225 (4)	165

Symmetry codes: (i) 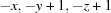; (ii) 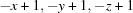; (iii) 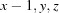; (iv) 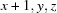; (v) 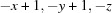; (vi) 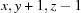; (vii) 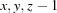; (viii) 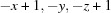.

**Table 3:** Experimental details

Crystal data	
Chemical formula	[Ni(C_19_H_13_N_3_O)_2_]Cl_2_·10H_2_O
*M*_r_	908.42
Crystal system, space group	Triclinic, *P* 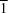
Temperature (K)	296
*a*, *b*, *c* (Å)	10.351 (7), 11.894 (8), 19.070 (13)
α, β, γ (°)	76.33 (1), 88.582 (12), 67.077 (11)
*V* (Å^3^)	2095 (2)
*Z*	2
Radiation type	Mo *K*α
μ (mm^−1^)	0.66
Crystal size (mm)	0.23 × 0.18 × 0.15

Data collection	
Diffractometer	Bruker SMART CCD area-detector
Absorption correction	Multi-scan (*SADABS*; Bruker, 2012 ▸)
*T*_min_, *T*_max_	0.864, 0.908
No. of measured, independent and observed [*I* > 2σ(*I*)] reflections	10779, 7382, 5322
*R*_int_	0.029
(sin θ/λ)_max_ (Å^−1^)	0.597

Refinement	
*R*[*F* ^2^ > 2σ(*F* ^2^)], *wR*(*F* ^2^), *S*	0.050, 0.144, 1.07
No. of reflections	7382
No. of parameters	546
No. of restraints	75
H-atom treatment	H-atom parameters constrained
Δρ_max_, Δρ_min_ (e Å^−3^)	0.42, −0.53

Computer programs: *SMART* and *SAINT* (Bruker, 2012 ▸), *SHELXT* (Sheldrick, 2015*a* ▸); *SHELXL2014* (Sheldrick, 2015*b* ▸), *OLEX2*(Dolomanov *et al.*, 2009 ▸), *DIAMOND* (Brandenburg & Putz, 2008 ▸) and *publCIF* (Westrip, 2010 ▸).

## supplementary crystallographic information

### Crystal data

**Table:**

[Ni(C_19_H_13_N_3_O)_2_]Cl_2_·10H_2_O	*Z* = 2
*M**_r_* = 908.42	*F*(000) = 948
Triclinic, *P*1	*D*_x_ = 1.440 Mg m^−^^3^
*a* = 10.351 (7) Å	Mo *K*α radiation, λ = 0.71073 Å
*b* = 11.894 (8) Å	Cell parameters from 3615 reflections
*c* = 19.070 (13) Å	θ = 2.2–24.0°
α = 76.33 (1)°	µ = 0.66 mm^−^^1^
β = 88.582 (12)°	*T* = 296 K
γ = 67.077 (11)°	Block, brown
*V* = 2095 (2) Å^3^	0.23 × 0.18 × 0.15 mm

### Data collection

**Table:**

Bruker SMART CCD area-detector diffractometer	5322 reflections with *I* > 2σ(*I*)
Radiation source: fine-focus sealed tube	*R*_int_ = 0.029
phi and ω scans	θ_max_ = 25.1°, θ_min_ = 1.9°
Absorption correction: multi-scan (SADABS; Bruker, 2012)	*h* = −12→8
*T*_min_ = 0.864, *T*_max_ = 0.908	*k* = −14→13
10779 measured reflections	*l* = −22→19
7382 independent reflections	

### Refinement

**Table:**

Refinement on *F*^2^	Primary atom site location: structure-invariant direct methods
Least-squares matrix: full	Secondary atom site location: difference Fourier map
*R*[*F*^2^ > 2σ(*F*^2^)] = 0.050	Hydrogen site location: mixed
*wR*(*F*^2^) = 0.144	H-atom parameters constrained
*S* = 1.07	*w* = 1/[σ^2^(*F*_o_^2^) + (0.0689*P*)^2^] where *P* = (*F*_o_^2^ + 2*F*_c_^2^)/3
7382 reflections	(Δ/σ)_max_ < 0.001
546 parameters	Δρ_max_ = 0.42 e Å^−^^3^
75 restraints	Δρ_min_ = −0.53 e Å^−^^3^

### Special details

**Table:**

Geometry. All esds (except the esd in the dihedral angle between two l.s. planes) are estimated using the full covariance matrix. The cell esds are taken into account individually in the estimation of esds in distances, angles and torsion angles; correlations between esds in cell parameters are only used when they are defined by crystal symmetry. An approximate (isotropic) treatment of cell esds is used for estimating esds involving l.s. planes.

### Fractional atomic coordinates and isotropic or equivalent isotropic displacement parameters (Å^2^)

**Table:**

	*x*	*y*	*z*	*U*_iso_*/*U*_eq_	Occ. (<1)
Ni1	0.97894 (4)	0.78241 (4)	0.74747 (2)	0.03697 (15)	
Cl1	0.30308 (10)	0.17463 (10)	0.94851 (6)	0.0626 (3)	
Cl2	0.28593 (15)	0.70315 (14)	0.37352 (7)	0.0975 (4)	
N1	0.9435 (3)	0.6158 (2)	0.77056 (14)	0.0395 (6)	
N2	1.0808 (3)	0.6936 (2)	0.84370 (14)	0.0371 (6)	
N3	1.0524 (3)	0.9124 (2)	0.76977 (14)	0.0402 (6)	
N4	1.1361 (3)	0.7137 (2)	0.67860 (15)	0.0404 (6)	
N5	0.8802 (3)	0.8743 (2)	0.65098 (14)	0.0363 (6)	
N6	0.7778 (3)	0.8893 (3)	0.77339 (14)	0.0399 (6)	
O1W	0.0399 (3)	0.1312 (3)	1.0094 (2)	0.1017 (12)	
H1WA	0.1069	0.1479	0.9865	0.153*	
H1WB	−0.0184	0.1283	0.9785	0.153*	
O1	1.3786 (3)	0.5497 (3)	1.07455 (16)	0.0771 (8)	
O2W	0.3405 (4)	0.3554 (3)	0.8020 (2)	0.1191 (14)	
H2WB	0.4256	0.3390	0.7997	0.179*	
H2WA	0.3280	0.3179	0.8431	0.179*	
O2	0.7323 (4)	1.0325 (5)	0.38766 (18)	0.0652 (12)	0.786 (13)
O2A	0.5318 (13)	1.1546 (17)	0.4459 (8)	0.0652 (12)	0.214 (13)
O3W	0.0344 (6)	0.4187 (5)	0.4364 (3)	0.161 (2)	
H3WC	0.1242	0.3849	0.4377	0.241*	
H3WA	−0.0004	0.4792	0.3967	0.241*	
O4W	0.2936 (5)	0.4343 (4)	0.4358 (2)	0.1321 (15)	
H4WB	0.2366	0.5098	0.4401	0.198*	
H4WA	0.3534	0.4658	0.4168	0.198*	
O5W	0.3714 (4)	0.7092 (4)	0.21731 (19)	0.1075 (12)	
H5WA	0.3229	0.7484	0.2484	0.161*	
H5WB	0.3079	0.7751	0.1866	0.161*	
O6W	0.8002 (4)	0.6442 (4)	0.3295 (2)	0.1187 (14)	
H6WC	0.7594	0.6559	0.2898	0.178*	
H6WA	0.7339	0.6982	0.3442	0.178*	
O7W	0.5809 (3)	0.6957 (3)	0.42459 (18)	0.0892 (10)	
H7WA	0.5069	0.6842	0.4137	0.134*	
H7WB	0.6169	0.6500	0.4680	0.134*	
O8W	0.9848 (5)	0.7051 (5)	0.4183 (3)	0.165 (2)	
H8WC	0.9741	0.6910	0.4634	0.247*	
H8WD	1.0541	0.7266	0.4086	0.247*	
O9W	0.1697 (4)	0.8720 (3)	0.08918 (18)	0.0972 (11)	
H9WA	0.2328	0.8596	0.0583	0.146*	
H9WB	0.1381	0.9527	0.0824	0.146*	
O10W	0.6127 (3)	0.1247 (3)	−0.00361 (17)	0.0790 (9)	
H10A	0.5244	0.1474	−0.0182	0.119*	
H10B	0.6177	0.0479	0.0111	0.119*	
C1	1.4491 (5)	0.4603 (6)	1.1365 (3)	0.0893 (16)	
H1A	1.4970	0.4732	1.1724	0.107*	
C2	1.4381 (5)	0.3551 (6)	1.1366 (2)	0.0892 (16)	
H2A	1.4785	0.2800	1.1719	0.107*	
C3	1.3555 (4)	0.3735 (4)	1.0750 (2)	0.0629 (10)	
H3A	1.3284	0.3149	1.0618	0.075*	
C4	1.3234 (4)	0.4928 (4)	1.03888 (19)	0.0502 (8)	
C5	1.2401 (3)	0.5626 (3)	0.97208 (18)	0.0439 (8)	
C6	1.2338 (3)	0.6820 (3)	0.93761 (18)	0.0450 (8)	
H6	1.2842	0.7184	0.9575	0.054*	
C7	1.1634 (3)	0.5118 (3)	0.93943 (18)	0.0435 (8)	
H7	1.1657	0.4322	0.9610	0.052*	
C8	1.0858 (3)	0.5796 (3)	0.87613 (17)	0.0400 (7)	
C9	1.1521 (3)	0.7449 (3)	0.87409 (17)	0.0386 (7)	
C10	1.1305 (3)	0.8734 (3)	0.83215 (18)	0.0405 (8)	
C11	1.1802 (4)	0.9497 (4)	0.8570 (2)	0.0519 (9)	
H11	1.2359	0.9201	0.9003	0.062*	
C12	1.1457 (4)	1.0711 (4)	0.8163 (2)	0.0591 (10)	
H12	1.1772	1.1250	0.8319	0.071*	
C13	1.0652 (4)	1.1115 (4)	0.7531 (2)	0.0574 (10)	
H13	1.0410	1.1932	0.7251	0.069*	
C14	1.0200 (4)	1.0298 (3)	0.7311 (2)	0.0506 (9)	
H14	0.9649	1.0577	0.6877	0.061*	
C15	0.9995 (3)	0.5383 (3)	0.83507 (18)	0.0384 (7)	
C16	0.9774 (4)	0.4297 (3)	0.8607 (2)	0.0485 (9)	
H16	1.0166	0.3774	0.9060	0.058*	
C17	0.8971 (4)	0.3996 (4)	0.8187 (2)	0.0568 (10)	
H17	0.8794	0.3274	0.8356	0.068*	
C18	0.8430 (4)	0.4757 (4)	0.7518 (2)	0.0557 (10)	
H18	0.7900	0.4550	0.7223	0.067*	
C19	0.8678 (4)	0.5830 (3)	0.7287 (2)	0.0491 (9)	
H19	0.8315	0.6347	0.6828	0.059*	
C20	1.2646 (4)	0.6268 (3)	0.6978 (2)	0.0507 (9)	
H20	1.2924	0.5921	0.7468	0.061*	
C21	1.3586 (4)	0.5861 (4)	0.6483 (2)	0.0624 (11)	
H21	1.4483	0.5246	0.6635	0.075*	
C22	1.3193 (4)	0.6362 (4)	0.5779 (2)	0.0605 (10)	
H22	1.3821	0.6104	0.5435	0.073*	
C23	1.1853 (4)	0.7262 (4)	0.5561 (2)	0.0515 (9)	
H23	1.1565	0.7615	0.5073	0.062*	
C24	1.0956 (3)	0.7623 (3)	0.60840 (18)	0.0389 (7)	
C25	0.9482 (3)	0.8536 (3)	0.59157 (17)	0.0383 (7)	
C26	0.8800 (4)	0.9120 (3)	0.52461 (18)	0.0438 (8)	
H26	0.9271	0.8958	0.4836	0.053*	
C27	0.7407 (4)	0.9953 (3)	0.51755 (18)	0.0421 (8)	
C28	0.6734 (4)	1.0179 (3)	0.57974 (18)	0.0423 (8)	
H28	0.5803	1.0746	0.5767	0.051*	
C29	0.7472 (3)	0.9549 (3)	0.64582 (17)	0.0367 (7)	
C30	0.6892 (3)	0.9652 (3)	0.71648 (17)	0.0378 (7)	
C31	0.5555 (4)	1.0467 (3)	0.7236 (2)	0.0488 (8)	
H31	0.4960	1.0997	0.6831	0.059*	
C32	0.5115 (4)	1.0483 (4)	0.7922 (2)	0.0551 (10)	
H32	0.4211	1.1021	0.7987	0.066*	
C33	0.6021 (4)	0.9698 (4)	0.8509 (2)	0.0549 (10)	
H33	0.5743	0.9694	0.8977	0.066*	
C34	0.7340 (4)	0.8921 (3)	0.83933 (19)	0.0499 (9)	
H34	0.7956	0.8391	0.8791	0.060*	
C35	0.6651 (4)	1.0581 (3)	0.44799 (17)	0.0473 (8)	
C36	0.5368 (6)	1.1455 (5)	0.4263 (4)	0.0530 (14)	0.786 (13)
H36	0.4693	1.1807	0.4566	0.064*	0.786 (13)
C36A	0.695 (2)	1.046 (2)	0.3801 (7)	0.0530 (14)	0.214 (13)
H36A	0.7779	0.9894	0.3672	0.064*	0.214 (13)
C37	0.5193 (7)	1.1758 (6)	0.3518 (4)	0.0582 (16)	0.786 (13)
H37	0.4390	1.2338	0.3231	0.070*	0.786 (13)
C37A	0.585 (3)	1.130 (2)	0.3344 (7)	0.0582 (16)	0.214 (13)
H37A	0.5793	1.1393	0.2846	0.070*	0.214 (13)
C38	0.6383 (8)	1.1068 (6)	0.3293 (2)	0.0593 (15)	0.786 (13)
H38	0.6561	1.1081	0.2811	0.071*	0.786 (13)
C38A	0.4838 (19)	1.198 (2)	0.3714 (10)	0.0593 (15)	0.214 (13)
H38A	0.3980	1.2620	0.3516	0.071*	0.214 (13)

### Atomic displacement parameters (Å^2^)

**Table:**

	*U*^11^	*U*^22^	*U*^33^	*U*^12^	*U*^13^	*U*^23^
Ni1	0.0399 (3)	0.0386 (3)	0.0332 (2)	−0.01552 (19)	0.00434 (16)	−0.01032 (18)
Cl1	0.0538 (6)	0.0650 (6)	0.0640 (6)	−0.0216 (5)	−0.0007 (4)	−0.0095 (5)
Cl2	0.1043 (10)	0.1146 (11)	0.0714 (9)	−0.0447 (9)	0.0106 (7)	−0.0168 (8)
N1	0.0398 (15)	0.0438 (16)	0.0373 (16)	−0.0172 (13)	0.0044 (12)	−0.0131 (13)
N2	0.0400 (15)	0.0369 (15)	0.0358 (15)	−0.0158 (12)	0.0047 (11)	−0.0110 (12)
N3	0.0430 (16)	0.0411 (16)	0.0405 (16)	−0.0189 (13)	0.0082 (12)	−0.0138 (13)
N4	0.0399 (16)	0.0407 (15)	0.0411 (17)	−0.0147 (13)	0.0069 (12)	−0.0136 (13)
N5	0.0383 (15)	0.0357 (14)	0.0361 (15)	−0.0151 (12)	0.0085 (11)	−0.0106 (12)
N6	0.0434 (15)	0.0457 (16)	0.0337 (15)	−0.0192 (13)	0.0088 (12)	−0.0132 (13)
O1W	0.075 (2)	0.106 (3)	0.121 (3)	−0.032 (2)	0.034 (2)	−0.033 (2)
O1	0.080 (2)	0.091 (2)	0.0604 (19)	−0.0284 (18)	−0.0039 (15)	−0.0255 (17)
O2W	0.153 (4)	0.072 (2)	0.096 (3)	−0.015 (2)	0.047 (3)	−0.008 (2)
O2	0.054 (2)	0.083 (3)	0.0378 (19)	−0.010 (2)	−0.0021 (15)	−0.0050 (18)
O2A	0.054 (2)	0.083 (3)	0.0378 (19)	−0.010 (2)	−0.0021 (15)	−0.0050 (18)
O3W	0.202 (5)	0.220 (6)	0.127 (4)	−0.143 (5)	0.047 (4)	−0.068 (4)
O4W	0.120 (3)	0.150 (4)	0.113 (4)	−0.051 (3)	−0.009 (3)	−0.011 (3)
O5W	0.149 (4)	0.128 (3)	0.073 (2)	−0.083 (3)	0.012 (2)	−0.025 (2)
O6W	0.114 (3)	0.157 (4)	0.141 (4)	−0.090 (3)	0.056 (3)	−0.079 (3)
O7W	0.085 (2)	0.081 (2)	0.084 (2)	−0.0156 (18)	−0.0003 (17)	−0.0184 (18)
O8W	0.163 (5)	0.214 (6)	0.121 (4)	−0.081 (4)	0.011 (3)	−0.034 (4)
O9W	0.097 (2)	0.104 (3)	0.092 (3)	−0.049 (2)	0.031 (2)	−0.015 (2)
O10W	0.0620 (18)	0.094 (2)	0.085 (2)	−0.0325 (17)	−0.0038 (15)	−0.0244 (18)
C1	0.064 (3)	0.129 (5)	0.055 (3)	−0.010 (3)	−0.018 (2)	−0.034 (3)
C2	0.089 (4)	0.093 (4)	0.042 (2)	0.003 (3)	−0.0113 (18)	−0.003 (2)
C3	0.075 (3)	0.0587 (19)	0.050 (2)	−0.022 (2)	0.0019 (17)	−0.0116 (17)
C4	0.047 (2)	0.0585 (18)	0.0374 (19)	−0.0128 (18)	0.0000 (14)	−0.0124 (14)
C5	0.0400 (19)	0.048 (2)	0.0362 (19)	−0.0093 (16)	0.0054 (14)	−0.0112 (16)
C6	0.0435 (19)	0.051 (2)	0.044 (2)	−0.0188 (17)	0.0034 (15)	−0.0183 (17)
C7	0.050 (2)	0.0385 (19)	0.0375 (19)	−0.0151 (16)	0.0043 (15)	−0.0055 (15)
C8	0.0401 (18)	0.0429 (19)	0.0377 (19)	−0.0152 (16)	0.0080 (14)	−0.0135 (16)
C9	0.0388 (18)	0.0428 (19)	0.0337 (18)	−0.0132 (15)	0.0066 (13)	−0.0141 (15)
C10	0.0414 (18)	0.0447 (19)	0.042 (2)	−0.0201 (16)	0.0089 (14)	−0.0181 (16)
C11	0.055 (2)	0.058 (2)	0.052 (2)	−0.0278 (19)	0.0027 (17)	−0.0200 (19)
C12	0.064 (3)	0.056 (2)	0.073 (3)	−0.034 (2)	0.014 (2)	−0.028 (2)
C13	0.071 (3)	0.043 (2)	0.063 (3)	−0.027 (2)	0.007 (2)	−0.0150 (19)
C14	0.055 (2)	0.045 (2)	0.048 (2)	−0.0179 (18)	0.0037 (16)	−0.0072 (17)
C15	0.0359 (17)	0.0386 (18)	0.0415 (19)	−0.0134 (15)	0.0070 (14)	−0.0139 (15)
C16	0.048 (2)	0.041 (2)	0.054 (2)	−0.0163 (17)	0.0054 (16)	−0.0071 (17)
C17	0.059 (2)	0.046 (2)	0.073 (3)	−0.0288 (19)	0.007 (2)	−0.016 (2)
C18	0.054 (2)	0.055 (2)	0.068 (3)	−0.029 (2)	0.0026 (19)	−0.022 (2)
C19	0.051 (2)	0.053 (2)	0.047 (2)	−0.0222 (18)	−0.0010 (16)	−0.0158 (18)
C20	0.044 (2)	0.049 (2)	0.056 (2)	−0.0134 (18)	0.0049 (17)	−0.0156 (18)
C21	0.038 (2)	0.064 (3)	0.077 (3)	−0.0086 (19)	0.0091 (19)	−0.023 (2)
C22	0.050 (2)	0.067 (3)	0.066 (3)	−0.018 (2)	0.024 (2)	−0.030 (2)
C23	0.050 (2)	0.059 (2)	0.047 (2)	−0.0196 (19)	0.0147 (16)	−0.0205 (18)
C24	0.0402 (18)	0.0378 (18)	0.043 (2)	−0.0171 (15)	0.0103 (14)	−0.0145 (15)
C25	0.0433 (19)	0.0414 (18)	0.0358 (18)	−0.0197 (16)	0.0114 (14)	−0.0154 (15)
C26	0.051 (2)	0.049 (2)	0.0360 (19)	−0.0231 (18)	0.0153 (15)	−0.0148 (16)
C27	0.050 (2)	0.0430 (19)	0.0353 (19)	−0.0212 (17)	0.0044 (14)	−0.0087 (15)
C28	0.0429 (19)	0.0409 (19)	0.040 (2)	−0.0133 (16)	0.0039 (14)	−0.0102 (15)
C29	0.0415 (19)	0.0349 (17)	0.0358 (18)	−0.0157 (15)	0.0086 (14)	−0.0121 (14)
C30	0.0422 (19)	0.0366 (18)	0.0377 (19)	−0.0169 (15)	0.0076 (14)	−0.0128 (15)
C31	0.047 (2)	0.047 (2)	0.048 (2)	−0.0144 (17)	0.0088 (16)	−0.0121 (17)
C32	0.051 (2)	0.059 (2)	0.060 (3)	−0.0197 (19)	0.0225 (19)	−0.027 (2)
C33	0.063 (2)	0.063 (2)	0.044 (2)	−0.025 (2)	0.0233 (18)	−0.023 (2)
C34	0.061 (2)	0.056 (2)	0.0343 (19)	−0.0238 (19)	0.0103 (16)	−0.0136 (17)
C35	0.058 (2)	0.057 (2)	0.0349 (19)	−0.029 (2)	0.0046 (16)	−0.0142 (17)
C36	0.057 (3)	0.050 (3)	0.049 (3)	−0.016 (2)	0.016 (3)	−0.019 (3)
C36A	0.057 (3)	0.050 (3)	0.049 (3)	−0.016 (2)	0.016 (3)	−0.019 (3)
C37	0.045 (3)	0.057 (3)	0.060 (3)	−0.010 (3)	−0.003 (3)	−0.010 (3)
C37A	0.045 (3)	0.057 (3)	0.060 (3)	−0.010 (3)	−0.003 (3)	−0.010 (3)
C38	0.056 (3)	0.074 (4)	0.033 (2)	−0.014 (3)	−0.004 (2)	−0.005 (2)
C38A	0.056 (3)	0.074 (4)	0.033 (2)	−0.014 (3)	−0.004 (2)	−0.005 (2)

### Geometric parameters (Å, º)

**Table:**

Ni1—N5	1.974 (3)	C7—H7	0.9300
Ni1—N2	1.977 (3)	C8—C15	1.487 (4)
Ni1—N6	2.093 (3)	C9—C10	1.480 (4)
Ni1—N3	2.096 (3)	C10—C11	1.376 (5)
Ni1—N1	2.098 (3)	C11—C12	1.378 (5)
Ni1—N4	2.099 (3)	C11—H11	0.9300
N1—C15	1.335 (4)	C12—C13	1.356 (6)
N1—C19	1.350 (4)	C12—H12	0.9300
N2—C8	1.331 (4)	C13—C14	1.377 (5)
N2—C9	1.339 (4)	C13—H13	0.9300
N3—C14	1.329 (4)	C14—H14	0.9300
N3—C10	1.333 (4)	C15—C16	1.373 (5)
N4—C20	1.322 (4)	C16—C17	1.364 (5)
N4—C24	1.332 (4)	C16—H16	0.9300
N5—C29	1.326 (4)	C17—C18	1.361 (5)
N5—C25	1.340 (4)	C17—H17	0.9300
N6—C34	1.329 (4)	C18—C19	1.368 (5)
N6—C30	1.332 (4)	C18—H18	0.9300
O1W—H1WA	0.8732	C19—H19	0.9300
O1W—H1WB	0.8701	C20—C21	1.370 (5)
O1—C4	1.336 (4)	C20—H20	0.9300
O1—C1	1.379 (6)	C21—C22	1.336 (6)
O2W—H2WB	0.8275	C21—H21	0.9300
O2W—H2WA	0.8393	C22—C23	1.379 (5)
O2—C35	1.364 (4)	C22—H22	0.9300
O2—C38	1.373 (5)	C23—C24	1.373 (4)
O2A—C35	1.404 (9)	C23—H23	0.9300
O2A—C38A	1.421 (9)	C24—C25	1.476 (5)
O3W—H3WC	0.8556	C25—C26	1.362 (5)
O3W—H3WA	0.8797	C26—C27	1.383 (5)
O4W—H4WB	0.8839	C26—H26	0.9300
O4W—H4WA	0.8729	C27—C28	1.389 (4)
O5W—H5WA	0.8692	C27—C35	1.435 (5)
O5W—H5WB	0.8890	C28—C29	1.374 (5)
O6W—H6WC	0.8315	C28—H28	0.9300
O6W—H6WA	0.8339	C29—C30	1.474 (4)
O7W—H7WA	0.8667	C30—C31	1.372 (5)
O7W—H7WB	0.8744	C31—C32	1.375 (5)
O8W—H8WC	0.8502	C31—H31	0.9300
O8W—H8WD	0.8528	C32—C33	1.372 (5)
O9W—H9WA	0.8616	C32—H32	0.9300
O9W—H9WB	0.8629	C33—C34	1.365 (5)
O10W—H10A	0.8785	C33—H33	0.9300
O10W—H10B	0.8705	C34—H34	0.9300
C1—C2	1.299 (7)	C35—C36	1.328 (6)
C1—H1A	0.9300	C35—C36A	1.352 (9)
C2—C3	1.394 (6)	C36—C37	1.378 (6)
C2—H2A	0.9300	C36—H36	0.9300
C3—C4	1.334 (5)	C36A—C37A	1.344 (10)
C3—H3A	0.9300	C36A—H36A	0.9300
C4—C5	1.432 (5)	C37—C38	1.315 (6)
C5—C6	1.394 (5)	C37—H37	0.9300
C5—C7	1.400 (5)	C37A—C38A	1.351 (9)
C6—C9	1.368 (5)	C37A—H37A	0.9300
C6—H6	0.9300	C38—H38	0.9300
C7—C8	1.355 (5)	C38A—H38A	0.9300
			
N5—Ni1—N2	178.36 (10)	N3—C14—H14	118.9
N5—Ni1—N6	77.81 (11)	C13—C14—H14	118.9
N2—Ni1—N6	102.65 (11)	N1—C15—C16	121.9 (3)
N5—Ni1—N3	100.71 (11)	N1—C15—C8	114.7 (3)
N2—Ni1—N3	77.74 (11)	C16—C15—C8	123.5 (3)
N6—Ni1—N3	89.84 (11)	C17—C16—C15	119.0 (4)
N5—Ni1—N1	103.80 (10)	C17—C16—H16	120.5
N2—Ni1—N1	77.77 (11)	C15—C16—H16	120.5
N6—Ni1—N1	93.13 (11)	C18—C17—C16	119.8 (3)
N3—Ni1—N1	155.38 (11)	C18—C17—H17	120.1
N5—Ni1—N4	78.10 (11)	C16—C17—H17	120.1
N2—Ni1—N4	101.46 (12)	C17—C18—C19	119.1 (3)
N6—Ni1—N4	155.89 (11)	C17—C18—H18	120.5
N3—Ni1—N4	95.46 (11)	C19—C18—H18	120.5
N1—Ni1—N4	91.74 (10)	N1—C19—C18	121.7 (4)
C15—N1—C19	118.5 (3)	N1—C19—H19	119.1
C15—N1—Ni1	114.5 (2)	C18—C19—H19	119.1
C19—N1—Ni1	126.9 (2)	N4—C20—C21	122.5 (4)
C8—N2—C9	120.0 (3)	N4—C20—H20	118.8
C8—N2—Ni1	120.1 (2)	C21—C20—H20	118.8
C9—N2—Ni1	119.7 (2)	C22—C21—C20	118.9 (4)
C14—N3—C10	118.6 (3)	C22—C21—H21	120.6
C14—N3—Ni1	126.4 (2)	C20—C21—H21	120.6
C10—N3—Ni1	114.8 (2)	C21—C22—C23	119.9 (4)
C20—N4—C24	118.8 (3)	C21—C22—H22	120.0
C20—N4—Ni1	127.0 (3)	C23—C22—H22	120.0
C24—N4—Ni1	114.1 (2)	C24—C23—C22	118.4 (4)
C29—N5—C25	120.9 (3)	C24—C23—H23	120.8
C29—N5—Ni1	119.5 (2)	C22—C23—H23	120.8
C25—N5—Ni1	119.6 (2)	N4—C24—C23	121.5 (3)
C34—N6—C30	118.6 (3)	N4—C24—C25	115.4 (3)
C34—N6—Ni1	126.8 (2)	C23—C24—C25	123.1 (3)
C30—N6—Ni1	114.5 (2)	N5—C25—C26	120.4 (3)
H1WA—O1W—H1WB	109.3	N5—C25—C24	112.8 (3)
C4—O1—C1	105.9 (4)	C26—C25—C24	126.8 (3)
H2WB—O2W—H2WA	108.4	C25—C26—C27	120.0 (3)
C35—O2—C38	106.7 (4)	C25—C26—H26	120.0
C35—O2A—C38A	104.3 (8)	C27—C26—H26	120.0
H3WC—O3W—H3WA	110.8	C26—C27—C28	118.7 (3)
H4WB—O4W—H4WA	89.3	C26—C27—C35	121.7 (3)
H5WA—O5W—H5WB	81.1	C28—C27—C35	119.7 (3)
H6WC—O6W—H6WA	96.0	C29—C28—C27	118.6 (3)
H7WA—O7W—H7WB	110.0	C29—C28—H28	120.7
H8WC—O8W—H8WD	111.3	C27—C28—H28	120.7
H9WA—O9W—H9WB	101.0	N5—C29—C28	121.4 (3)
H10A—O10W—H10B	87.7	N5—C29—C30	113.5 (3)
C2—C1—O1	109.2 (4)	C28—C29—C30	125.1 (3)
C2—C1—H1A	125.4	N6—C30—C31	122.3 (3)
O1—C1—H1A	125.4	N6—C30—C29	114.5 (3)
C1—C2—C3	108.6 (5)	C31—C30—C29	123.1 (3)
C1—C2—H2A	125.7	C30—C31—C32	118.4 (3)
C3—C2—H2A	125.7	C30—C31—H31	120.8
C4—C3—C2	105.5 (4)	C32—C31—H31	120.8
C4—C3—H3A	127.3	C33—C32—C31	119.4 (3)
C2—C3—H3A	127.3	C33—C32—H32	120.3
C3—C4—O1	110.8 (3)	C31—C32—H32	120.3
C3—C4—C5	129.7 (4)	C34—C33—C32	118.7 (3)
O1—C4—C5	119.5 (3)	C34—C33—H33	120.7
C6—C5—C7	118.2 (3)	C32—C33—H33	120.7
C6—C5—C4	121.2 (3)	N6—C34—C33	122.6 (3)
C7—C5—C4	120.6 (3)	N6—C34—H34	118.7
C9—C6—C5	118.8 (3)	C33—C34—H34	118.7
C9—C6—H6	120.6	C36—C35—O2	107.6 (4)
C5—C6—H6	120.6	C36A—C35—O2A	109.5 (8)
C8—C7—C5	119.4 (3)	C36—C35—C27	133.6 (4)
C8—C7—H7	120.3	C36A—C35—C27	133.4 (8)
C5—C7—H7	120.3	O2—C35—C27	118.8 (3)
N2—C8—C7	121.8 (3)	O2A—C35—C27	117.1 (7)
N2—C8—C15	112.6 (3)	C35—C36—C37	109.4 (4)
C7—C8—C15	125.6 (3)	C35—C36—H36	125.3
N2—C9—C6	121.7 (3)	C37—C36—H36	125.3
N2—C9—C10	112.7 (3)	C37A—C36A—C35	108.2 (9)
C6—C9—C10	125.5 (3)	C37A—C36A—H36A	125.9
N3—C10—C11	122.2 (3)	C35—C36A—H36A	125.9
N3—C10—C9	114.7 (3)	C38—C37—C36	106.7 (4)
C11—C10—C9	122.9 (3)	C38—C37—H37	126.7
C10—C11—C12	118.4 (4)	C36—C37—H37	126.7
C10—C11—H11	120.8	C36A—C37A—C38A	110.1 (10)
C12—C11—H11	120.8	C36A—C37A—H37A	124.9
C13—C12—C11	119.5 (3)	C38A—C37A—H37A	124.9
C13—C12—H12	120.3	C37—C38—O2	109.6 (4)
C11—C12—H12	120.3	C37—C38—H38	125.2
C12—C13—C14	119.1 (4)	O2—C38—H38	125.2
C12—C13—H13	120.4	C37A—C38A—O2A	107.8 (9)
C14—C13—H13	120.4	C37A—C38A—H38A	126.1
N3—C14—C13	122.1 (4)	O2A—C38A—H38A	126.1

### Hydrogen-bond geometry (Å, º)

**Table:**

*D*—H···*A*	*D*—H	H···*A*	*D*···*A*	*D*—H···*A*
O1*W*—H1*WA*···Cl1	0.87	2.25	3.113 (4)	169
O1*W*—H1*WB*···O9*W*^i^	0.87	2.06	2.923 (6)	175
O2*W*—H2*WB*···O5*W*^ii^	0.83	1.99	2.813 (7)	172
O2*W*—H2*WA*···Cl1	0.84	2.39	3.215 (4)	168
O3*W*—H3*WC*···O4*W*	0.86	2.05	2.760 (9)	140
O3*W*—H3*WA*···O6*W*^iii^	0.88	2.35	3.134 (7)	148
O4*W*—H4*WB*···Cl2	0.88	2.58	3.107 (5)	119
O4*W*—H4*WA*···Cl2	0.87	2.56	3.107 (5)	122
O5*W*—H5*WA*···Cl2	0.87	2.37	3.079 (4)	138
O5*W*—H5*WB*···O9*W*	0.89	2.16	2.991 (6)	156
O6*W*—H6*WC*···O2*W*^ii^	0.83	2.11	2.929 (6)	167
O6*W*—H6*WA*···O7*W*	0.83	2.18	2.838 (6)	136
O7*W*—H7*WA*···Cl2	0.87	2.34	3.190 (4)	167
O7*W*—H7*WB*···O4*W*^ii^	0.87	1.93	2.798 (5)	172
O8*W*—H8*WC*···O3*W*^ii^	0.85	2.06	2.856 (8)	155
O8*W*—H8*WD*···Cl2^iv^	0.85	2.40	3.204 (6)	157
O9*W*—H9*WA*···O10*W*^v^	0.86	1.93	2.756 (6)	159
O9*W*—H9*WB*···O1*W*^vi^	0.86	2.11	2.878 (5)	147
O10*W*—H10*A*···Cl1^vii^	0.88	2.27	3.141 (4)	171
O10*W*—H10*B*···Cl1^viii^	0.87	2.38	3.225 (4)	165

Symmetry codes: (i) −*x*, −*y*+1, −*z*+1; (ii) −*x*+1, −*y*+1, −*z*+1; (iii) *x*−1, *y*, *z*; (iv) *x*+1, *y*, *z*; (v) −*x*+1, −*y*+1, −*z*; (vi) *x*, *y*+1, *z*−1; (vii) *x*, *y*, *z*−1; (viii) −*x*+1, −*y*, −*z*+1.
